# DIGGER-Bac: prediction of seed regions for high-fidelity construction of synthetic small RNAs in bacteria

**DOI:** 10.1093/bioinformatics/btad285

**Published:** 2023-04-22

**Authors:** Niklas Philipp, Cedric K Brinkmann, Jens Georg, Daniel Schindler, Bork A Berghoff

**Affiliations:** Institute for Microbiology and Molecular Biology, Justus-Liebig University Giessen, Giessen 35392, Germany; Max-Planck-Institute for Terrestrial Microbiology, Marburg 35043, Germany; Institut für Biologie III, Albert-Ludwigs-Universität Freiburg, Freiburg 79104, Germany; Max-Planck-Institute for Terrestrial Microbiology, Marburg 35043, Germany; Center for Synthetic Microbiology (SYNMIKRO), Philipps-University Marburg, Marburg 35043, Germany; Institute for Microbiology and Molecular Biology, Justus-Liebig University Giessen, Giessen 35392, Germany

## Abstract

Synthetic small RNAs (sRNAs) are gaining increasing attention in the field of synthetic biology and bioengineering for efficient post-transcriptional regulation of gene expression. However, the optimal design of synthetic sRNAs is challenging because alterations may impair functions or off-target effects can arise. Here, we introduce DIGGER-Bac, a toolbox for Design and Identification of seed regions for Golden Gate assembly and Expression of synthetic sRNAs in Bacteria. The SEEDling tool predicts optimal sRNA seed regions in combination with user-defined sRNA scaffolds for efficient regulation of specified mRNA targets. Results are passed on to the G-GArden tool, which assists with primer design for high-fidelity Golden Gate assembly of the desired synthetic sRNA constructs.

## 1 Introduction

**Figure 1.**
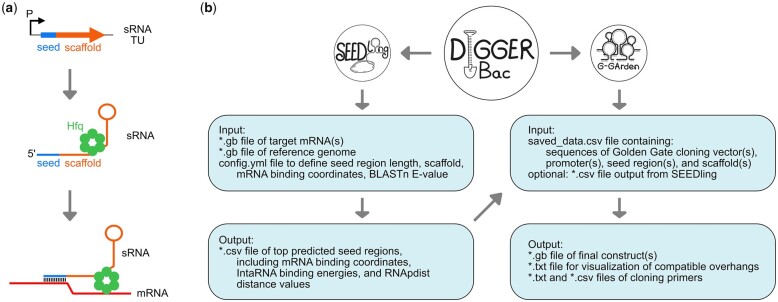
Prediction and design of synthetic sRNAs for post-transcriptional gene regulation using DIGGER-Bac. (a) Illustration of an sRNA TU and the resulting sRNA. The promoter (P), the sRNA seed region and the sRNA scaffold with terminator (lollipop structure), and a potential binding site for the hexameric RNA chaperone Hfq are indicated. The seed region binds by base-pairing to target mRNAs. (b) The DIGGER-Bac toolbox provides the SEEDling and G-GArden tools for seed prediction and optimal scaffold selection as well as primer design for Golden Gate cloning strategies, respectively. The SEEDling output may be applied as input for G-GArden. However, both tools can be used as stand-alone applications.

In bacteria, small RNAs (sRNAs) are versatile regulators that can be exploited for modulation of gene expression at the post-transcriptional level. Hence, sRNAs have gained attention as regulatory tools in bacteria using synthetic biology approaches (Na et al. 2013; Noh et al. 2017; Dwijayanti et al. 2022). A typical sRNA transcriptional unit (TU) consists of (i) a promoter, (ii) a sequence for the sRNA seed region, and (iii) a scaffold sequence (Fig. 1a). The seed region binds by base-pairing to target mRNAs to regulate translation and/or mRNA stability, and the scaffold provides important structural elements, such as RNA chaperone binding sites and a Rho-independent terminator (Fig. 1a) (Storz et al. 2011). The small size and modular structure of sRNA TUs enable easy and cost-effective construction of versatile and directed synthetic sRNA-based regulators (Köbel et al. 2022).

In recent years, type IIS restriction enzyme-based cloning, Golden Gate cloning, became popular because type IIS enzymes recognize specific sequences but cut in a directed and defined distance outside the motif, allowing scar-free assembly. Using Golden Gate assembly, several DNA parts can be combined in a one-pot, single-step reaction with high precision (Engler et al. 2008). The method relies on short single-stranded overhangs with a size of 3 or 4 base pairs. User-defined overhangs enable assembly of DNA parts in a controlled manner. When combining several parts in one-pot reactions, it is important to use Golden Gate-compatible cloning plasmids and unique overhangs that allow DNA assembly with high fidelity (Potapov et al. 2018a, 2018b).

We have recently established an easy-to-use plasmid toolset for efficient Golden Gate-based generation of synthetic sRNA TUs in *Escherichia coli* (Köbel et al. 2022). The plasmid tool set can be quickly adapted to other model bacteria and, therefore, represents a versatile resource for synthetic sRNA construction. The design of seed regions and selection of scaffolds is a critical step in construction of effective sRNA regulators. The DIGGER-Bac (Design and Identification of seed regions for Golden Gate cloning and Expression of synthetic sRNAs in Bacteria) toolbox supports the process of seed region/scaffold identification (SEEDling) and guides the primer design for high-fidelity Golden Gate cloning (G-GArden) (Fig. 1b).

## 2 SEEDling: prediction of seed regions and scaffolds for efficient target regulation

SEEDling predicts the best possible synthetic sRNA sequence for the target(s) of choice. The lowest sRNA–mRNA hybridization energy does not necessarily provide the best function; therefore, SEEDling integrates hybridization energy, off-target activity, and sRNA structure to predict the optimal synthetic sRNA. In order to do so it requires the following user inputs: (i) an annotated target genbank file (single gene to whole genome), (ii) the reference genome of the used model organism, and (iii) a config file (i.e. to define seed region length, scaffold, mRNA binding coordinates, and E-value for BLASTn) (Fig. 1b). Based on the input files, SEEDling predicts the best antisense regions and uses BLASTn (Camacho et al. 2009) to determine potential off-target binding to the whole reference genome by the expect value (E-value). Afterwards, it combines the seed region with the provided sRNA scaffold to determine the binding energy via IntaRNA (Mann et al. 2017), and finally applies RNApdist (Lorenz et al. 2011) to compare synthetic sRNAs and the wild-type sRNA in regard to potential folding alterations, allowing exclusion of synthetic sRNAs with drastic structural changes. For manual curation, we recommend RNAfold (Lorenz et al. 2011) to visualize the structure of the synthetic sRNAs. In its default mode, SEEDling generates multiple seed regions per target and ranks them based on the best match position, off-target effects, and lowest predicted structural folding alteration. SEEDling can be operated as a command line tool on Linux platforms. Besides the standard version available at GitHub, SEEDling is further available as a containerized version via Docker, which enables its use with any operation system. The output of SEEDling can be directly used to order synthetic DNA sequences for subsequent cloning or feed into the Golden Gate assembly tool (G-GArden) within the DIGGER-Bac toolbox. Notably, SEEDling ignores sequences containing excluded DNA motifs (i.e. type IIS recognition sites) to be compatible with downstream Golden Gate assembly. An exemplary output file can be found in Supplementary Data S1.

## 3 G-GArden: primer design for high-fidelity Golden Gate assembly

The G-GArden tool is a stand-alone application for primer design in Golden Gate-based cloning strategies. It is implemented as a graphical user interface (GUI) that is launched by an executable file on both Linux and Windows operating systems. G-GArden is written in python and bundled separately for both operating systems using PyInstaller so it can be executed without time-consuming installation of interpreters or modules. To take advantage of the DIGGER-Bac toolbox results from SEEDling are preferably used as input (Fig. 1b). G-GArden retrieves sequence information from its *saved_data* file that is constantly expanded by the user (e.g. by feeding in SEEDling results and acceptor plasmids). Within the GUI, four DNA parts are typically selected for construction of an sRNA TU: (i) a Golden Gate-compatible cloning plasmid, (ii) a promoter, (iii) a seed sequence, and (iv) a scaffold sequence (Fig. 1b). By selecting more than four DNA parts, the tool enables the design of more complex sRNAs (e.g. sRNAs with two seed regions) or concatenation of multiple sRNA TUs. Furthermore, several sequences can be selected for each DNA part, resulting in parallelized and combinatorial design of cloning strategies. The individual Golden Gate-compatible plasmid has to match the type IIS restriction enzyme that is selected for the cloning procedure. DNA parts are generated either by annealing of oligodeoxynucleotides or by polymerase chain reaction (PCR), which is specified by the user. When PCR is selected, the *primer3* tool (https://github.com/primer3-org) may be applied for primer design (Untergasser et al. 2012). By default, G-GArden computes overhangs for a scarless assembly strategy (i.e. overhangs do not produce ‘scars’ in the resulting synthetic sRNAs). Overhangs are checked subsequently for duplicates and overhangs that might result in low-fidelity assembly. If such problems occur, the user is referred to the NEBridge GetSet™ Tool (https://ggtools.neb.com/getset/run.cgi) for optimization of overhangs (Potapov et al. 2018a, 2018b). Upon successful completion, the user can open the output directory and access (i) visualization files to check the overhang-guided assembly strategy (*.txt*), (ii) files for ordering of oligodeoxynucleotides (*.txt* and *.csv*), (iii) and genbank files of the final constructs for documentation purposes (*.gb*). Exemplary *saved_data* and output files can be found in Supplementary Data S2, as generated using the SEEDling output from Supplementary Data S1 as input for G-GArden.

## 4 Conclusions

The Design-Build-Test-Learn cycle is at the heart of synthetic biology approaches. The DIGGER-Bac toolbox offers user-friendly solutions for the Design-Build phase of synthetic sRNA-related projects. While existing tools compute complementary RNA sequences (i.e. seed regions) for target regulation and search for potential off-target effects (Romero et al. 2022), SEEDling further optimizes sRNA-based regulation strategies by calculating binding energies (IntaRNA) and sRNA structures (RNApdist) based on the desired scaffold. It should be noted, however, that SEEDling does not consider binding sites for RNA chaperones (e.g. Hfq or ProQ) for the calculation of binding energies. Consequently, SEEDling might underestimate the regulatory potential of some chaperone-dependent sRNAs. It will be a future task to experimentally address whether SEEDling performs equally well for different kinds of sRNA scaffolds.

Finally, G-Garden assists with primer design for cloning, which—together with our recently published plasmid tool set (Köbel et al. 2022)—speeds up construction of sRNA expression systems. In summary, the presented tools enable easy access to synthetic sRNA design and are expected to promote the use of synthetic sRNAs as regulatory tools in many molecular biology laboratories.

## Supplementary Material

btad285_Supplementary_Data

## Data Availability

Both SEEDling and G-GArden are freely available at https://github.com/DIGGER-Bac under the CC BY-NC-SA 4.0 license.
